# Leadership Styles and Organizational Citizenship Behavior for the Environment: The Mediating Role of Self-Efficacy and Psychological Ownership

**DOI:** 10.3389/fpsyg.2021.683101

**Published:** 2021-07-02

**Authors:** Irfan Ullah, Worakamol Wisetsri, Hao Wu, Syed Mehmood Ali Shah, Ali Abbas, Shahid Manzoor

**Affiliations:** ^1^School of Management and Economics, Beijing Institute of Technology, Beijing, China; ^2^Department of Manufacturing and Service Industry Management, Faculty of Business and Industrial Development, King Mongkut's University of Technology North Bangkok (KMUTNB), Bangkok, Thailand; ^3^Northeast Asian Research Center, Jilin University, Changchun, China; ^4^Business School, University of International Business and Economics, Beijing, China; ^5^Hailey College of Commerce, University of the Punjab, Lahore, Pakistan

**Keywords:** responsible leadership, inclusive leadership, authentic leadership, supportive leadership, self-efficacy, OCBE, psychological ownership

## Abstract

Change and environmental patterns are having an immense effect upon the world. Businesses, communities, and even individuals are struggling to perform their role within environmental protection. This paper investigates the role of leadership styles on organizational citizenship behavior for the environment (OCBE) directly and through the mediation of self-efficacy and psychological ownership. The survey technique was used to collect the data from Chinese banking, insurance, medicine, and teaching service sector employees for the current study. The reliability and validity of the scale items were tested. This study used AMOS-SEM for data analysis and testing the developed hypotheses. The empirical results confirmed that responsible, inclusive, authentic, and supportive leadership styles positively impact employees' OCBE. The results further confirm that self-efficacy and psychological ownership act as mediators between leadership and OCBE. The current study widens our understanding of leadership styles and their impact on OCBE, along with limitations associated with the study and future guidelines for investigators.

## Graphical Abstract

Graphical abstract shows the visual representation of direct and indirect relationships. 
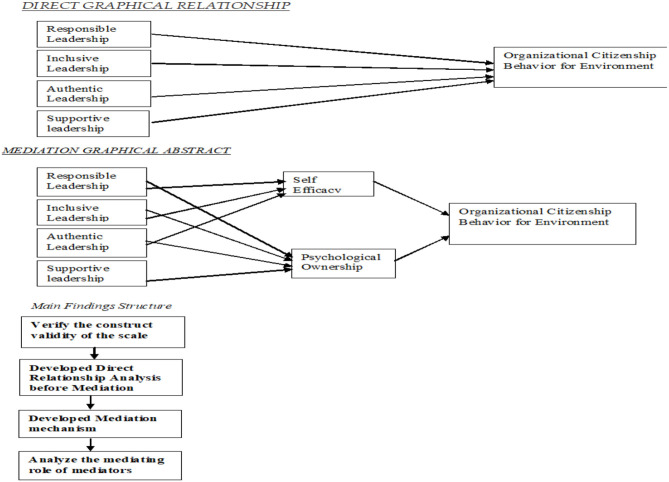

## Highlights

- The relationship between leadership styles and organizational citizenship behavior for the environment (OCBE) is quantitatively investigated.
- A significant relationship has been found through a direct relationship between leadership styles and OCBE.
- The mediation mechanism has been developed to analyze the mediating role of self-efficacy and psychological ownership.
- Self-efficacy partially mediates the relationship between leadership styles and OCBE.
- Psychological ownership partially mediates the relationship between leadership styles and OCBE.

## Introduction

As the world's environmental prospects worsen, and business management is shifting its attention to maintaining viable ecological development (Bansal and Song, 2017), several renowned researchers have put efforts into researching sustainable business management. They made efforts to check and balance the environmental shifting patterns at the strategic level but failed to pay attention at the worker level (Galpin and Whittington, 2012). This has attracted more attention and efforts at the worker level. Since workers execute business strategies, their outlook toward the environment plays a key role in supporting sustainable management at the business level (Felin et al., 2015). Under this reference, organizational citizenship behavior for the environment (OCBE) refers to the workers' environmental practices that are not demanded, awarded, or formally recognized by the organizations. These practices also complement the environmental defensive attitude of social inhabitants and the green development strategic business analysis. At work, workers engage in OCBE, strictly building their environmental defensive attitudes and concepts, thus fulfilling the organization's green strategic analysis and philosophy (Ramus and Steger, 2000). Thus, workers' OCBE has an important influence on company environmental presentation and practices (Paillé et al., 2013; Paillé and Mejía-Morelos, 2014; Lamm et al., 2015; Temminck et al., 2015). Moreover, based on past experiences, it can be concluded that workers engaging in OCBE are of great significance. The present-day management researchers are, remarkably, evolving their interests in establishing the workers' organizational sustenance and growth (Paillé and Mejía-Morelos, 2014), environmental self-caring (Zhang et al., 2016), business environmental security procedures (Paillé et al., 2013), business environmental problems (Temminck et al., 2015), and business environmental behaviors (Lamm et al., 2015). In the case of prevailing attitudes, the study authenticates moral leadership (Zhang et al., 2016) and the presidency as being sustenance for environmental security (Priyankara et al., 2018) that can increase workers' OCBE. Even though some researchers have examined the effect of leaders on OCBE, others discovered the impact of responsible leadership on OCBE. A surfeit of literature is available on leadership and OCBE, but little is known about the role of different leadership styles, which has not gained much attention. The current study is an attempt to fulfill this research gap. Likewise, responsible leadership pays attention to society, the environment, and supports beneficial change (Pless et al., 2011). Synchronized development among people, nature, and society is the central differential between careful leadership and other customary leadership ideas such as transformational, supportive, authentic, and service leadership.

Nevertheless, several studies have identified the impact of numerous leadership styles (e.g., transformational, supportive, authentic, and service) on OCBE as being responsible, inclusive, authentic but, more specifically, focused on society, for sustainable environmental development and positive change (Pless et al., 2011). Many enterprise researchers investigated environmental behavior at the strategic and management level of sustainable organizational development but overlooked it at the employees' level (Galpin and Whittington, 2012). A profound look at past studies highlighted the role of leadership in green behavior (Kura, 2016; Zhang et al., 2016). OCBEs are intensely focused on individual discretionary behavior. A formal reward system is not directly recognized within the organization but is, more specifically, collectively, and immediately helping the natural environment and ultimately contributing to organizational sustainable development (Robertson and Barling, 2017).

Given the above discussion, two objectives can be evolved and are entailed by the current study. First, it examines how responsible, inclusive, authentic, and supportive leadership styles contribute to employees' OCBE. Second, it looks at the mediating role of self-efficacy and psychological ownership between the relationship of the leadership styles (responsible, inclusive, authentic, and supportive) and OCBE to support the green behavior of employees.

### Literature Review and Theorization of Hypotheses

#### Leadership Styles and OCBE

The concept of OCBEs was introduced in 2009 by Daily et al. (2009), who described it as *individual and discretionary social behaviors that are not explicitly recognized by the formal reward system and contribute to more effective environmental management organizations*. The concept of OCBE is based on the idea of organizational citizenship behavior (OCB) but is distinct from it (Lamm et al., 2013). They said that OCBE is a combination of three attributes: First, it happens in the office and workers initiate it. Second, these activities are governed by motivation, and third, these actions lead to environmental protection and benefit the organization's environmental success and sustainability (Lamm et al., 2013). The voluntary behavior of OCBE includes reduced consumption of energy and resources, less carbon footprinting, less usage of paper to save trees, helping colleagues, and proposing work suggestions in environment-friendly ways (Boiral and Paillé, 2012).

They further added that OCBE behavior works in three contexts, i.e., eco-initiatives, eco-helping, and eco-civic engagements. Eco-initiatives are self-initiated and initial steps that the individual takes to upkeep the environmental activities. Eco-helping describes a work setting in which colleagues help each other in such activities that are pro-environmental. Moreover, eco-civic engagements are green activities in the workplace. These include steps and actions that contribute to the environment. On the one hand, OCBE, with the interaction of formal environmental management systems, fills the environmental gaps that are not addressed by regulatory systems, while, on the other hand, it reduces the organizational environmental costs by enhancing its reputation in the arena of environmental protection (Paillé et al., 2014; Alt and Spitzeck, 2016; Zhang et al., 2016).

Leadership is one of the essential phenomena in management studies. It is considered a fundamental concept which implies influence over followers and subordinates and helps in organizational and management development theory. In a conceptualized domain, leadership falls within the behavioral realms. According to Goleman (2004), a great emphasis has been placed on leadership concepts and practices dominant in organizational settings. Leaders are committed to, and interested in, achieving organizational goals effectively and efficiently. Leaders or managers tend to continue learning leadership skills and characteristics to manage upcoming challenges. Unforeseen situations that could occur unexpectedly, and may arise in different circumstances, can only be overcome through leaders learning various skills. Leaders always assist their organizations in various challenging situations successfully; therefore, those organizations that do not have well-developed leaders may face challenges, even failure. Management researchers widely accept these considerations, that the term “leaders” does not exclusively mean a “born leader,” but they arrive at a conclusion that leadership skills and qualities could be learned and developed. Several researchers made efforts to develop different instruments to measure the multiple styles of leadership, behavioral traits, and set of skills associated with leadership styles (Rooney and Gottlieb, 2007; Walumbwa et al., 2008; Carmeli et al., 2010; Voegtlin, 2011).

Past studies have revealed several factors that influence OCBEs; more precisely, past studies have shown that leadership can significantly affect the employees' green behavior. Past literature also indicated the positive associations between leadership styles and green behavior (Robertson and Barling, 2013; Kura, 2016). These leadership styles are numerous, but it can be found that specific leadership styles can influence specific behaviors and intentions (Conchie et al., 2012). The roles and implications of leadership are vital for an organization and are examined and proved in several prior studies (Nembhard and Edmondson, 2006; Walumbwa et al., 2008). Hence, different leadership styles and their traits can affect other characteristics and aspects. The current study specifically examines the role of leadership styles and traits, particularly affecting the OCBEs, where employees have had a role in the success of an organization, specifically in its environmental performance (Boiral and Paillé, 2012; Chan and Hsu, 2016). This study investigated four leadership styles: responsible, inclusive, authentic, and supportive leadership. It strives to promote a new perspective for OCBE.

**Figure 1:**
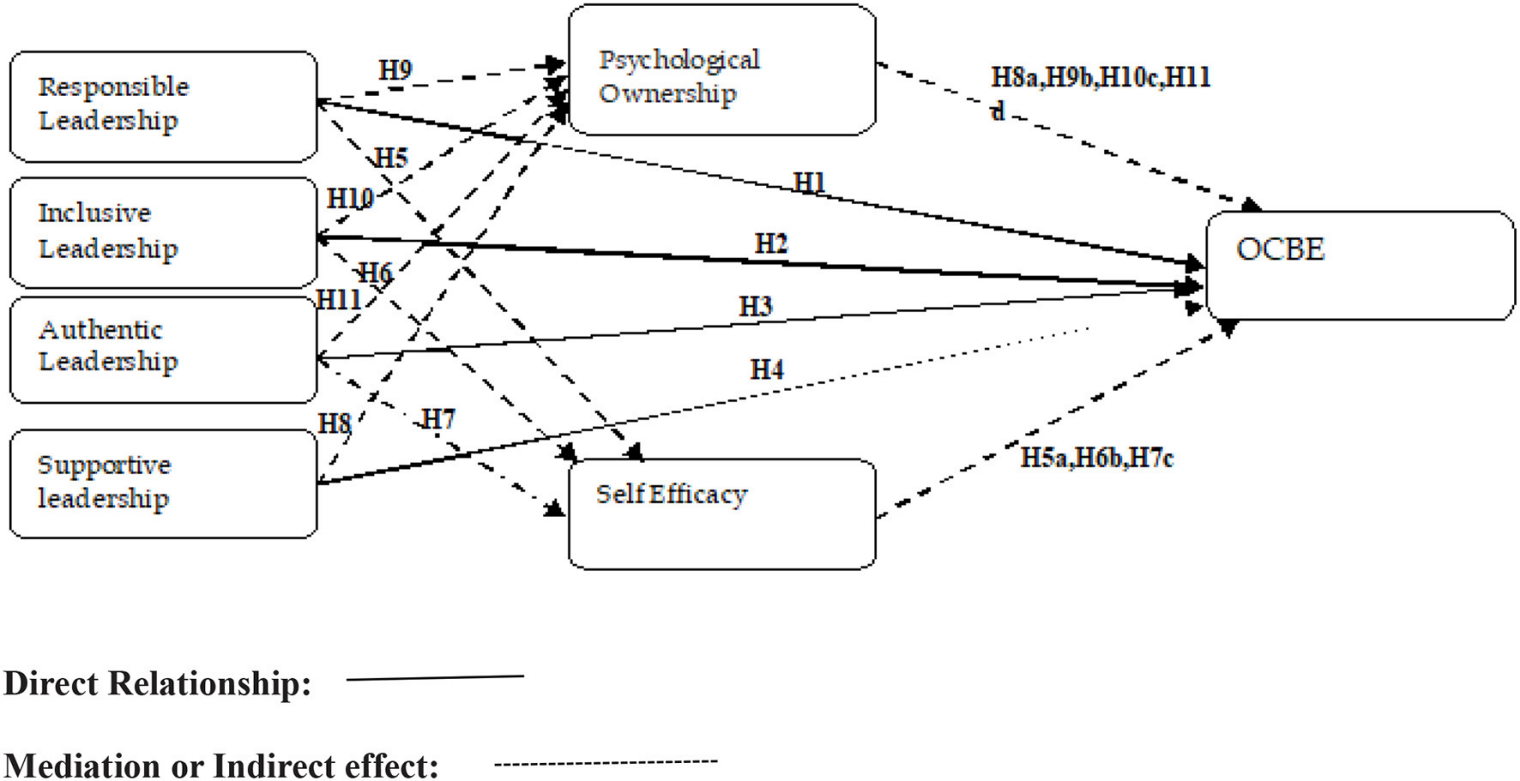
Research framework.

The research model is depicted in Figure 1.

#### Responsible Leaderships and OCBE

Responsible leadership is a set of behavioral traits that pay attention, not only to the interests of the business, but also to those of the stakeholders (Han et al., 2019a). Responsible leaders combine leadership traits with social responsibility by keeping in view the interests of various stakeholders of the organization. A responsible leader strives to integrate and protect the economic, social, and ecological interests and benefits of all the stakeholders, including employees (Han et al., 2019a; Zhao and Zhou, 2019). Responsible leadership places equal responsibility on all aspects of the organization, and takes justified decisions through rational analysis of the interests of all the stakeholders for sustainable development, trust-building, and green action choices. Responsible leaders can promote credible characteristics through their reputable actions and performance.

Employees in organizations with paper-saving behavior, lower energy consumption, enhanced environmental protection, giving assistance to others in practicing green behavior, and recommending enhanced environmental protection are typical examples of OCBE (Afsar et al., 2020). Social learning theory argues that individuals could learn through their behavior by observing others. Social learning theory also emphasizes that employees learn through focusing on a role model and adopt behaviors depending on what actions are rewarded (Bandura, 1986). Responsible leaders vie to integrate and save all the stakeholders' interests and benefits, particularly their ecological interests. This view is according to the scope of, and helps employees to engage in, the OCBE. Based on the above literature, the following hypothesis can be formulated:

***H1:** Responsible leadership has a significant relationship with OCBE*.

#### Inclusive Leadership and OCBE

The concept of inclusive leadership is defined as “the degree to which an employee perceives that he or she is an esteemed member of the workgroup through experiencing treatment that satisfies his or her needs for belongingness and uniqueness” (Shore et al., 2011). Inclusive leadership includes the people and employees in the affairs of the organization. Inclusive leadership refers to doing things with people instead of doing things to people. In this way, inclusive leadership creates a sense of belonging and uniqueness. Employees consider themselves included when they feel a higher sense of being an integral part of the organization, develop their association with the organization, make efforts to accomplish the tasks for mutual benefits, and strive for long-term results. Employees reported that their work with others is unique, that they feel valued because of their exceptional talent, and perceived themselves to belong to a distinguished team (Sugiyama et al., 2016). Compared with other leadership styles, inclusive leadership has a unique level of acceptance within organizational perspectives based on its uniqueness, inclusiveness, and sense of belonging. Optimal distinctiveness theory, an extension of social identity theory, discusses that individuals always need to be both different from, and similar to, others simultaneously.

The conceptualization of inclusion is viewed as the individual's need for a sense of belonging and uniqueness. Creating the state of gain, inclusive leadership simultaneously refers to both being different from, and similar to, the others (Randel et al., 2018). In this way, it facilitates a sense of belonging within the organization and its policies. Inclusive leaders focus on including all employees in the discussion and decision-making process and also value their voice. The shared decision-making process and inclusion in organizational discussion encourages environmentally oriented employees to promote and implement new ideas and establish new practices to save the environment. Employees need the support of organizations that they found in the shape of inclusive leadership, to execute innovative and environmentally friendly behavior (Javed et al., 2017). Similarly, based on the literary discussion and theoretical premises, we can assume that:

***H2:** Inclusive leadership has a significant relationship with OCBE*.

#### Authentic Leadership and OCBE

Authentic leadership is a branch of positive organizational behavior that claims that an authentic leader is aware of his strengths and weaknesses, encourages followers, and does not enforce his point of view on followers. His actions are according to personal values, feelings, and beliefs. Authentic leadership refers to the “behavior that draws upon and promotes both positive psychological capacities and a positive ethical climate. It fosters greater self-awareness, an internalized moral perspective, balanced processing of information, and relational transparency on the part of leaders working with followers, fostering positive self-development” (Walumbwa et al., 2008). Authentic leadership is a combination of four components, i.e., internalized morals, relational transparency, self-awareness, and balanced processing (Iqbal et al., 2018). Among other attributes, Avolio and Gardner (2005) consider a constructive moral perspective, leader self-regulation and awareness, follower self-regulation and understanding, and follower development very valuable in the organizational context.

The leadership role is vital in the development of employees' behavior and attitudes in the workplace. For the most part, authentic leadership plays a vital role in enhancing the voluntary behavior of employees in the workplace, i.e., OCB (Iqbal et al., 2018). The study of Liu et al. (2018) empirically proved that authentic leadership is significantly and positively related to the proactive behavior of subordinates. It also stimulates and upholds a positive and supportive climate, bringing transparency into a leader–employee relationship (Walumbwa et al., 2008). This helps employees develop self-awareness and positive behaviors, which are explicitly beneficial within organizational and societal contexts, citizenship, and environmental-protective behaviors. An authentic leader's behavior assists an open work environment and facilitates transparency that affects the employee's attitude and development toward satisfaction. Hence, in this study, we postulate that there is a positive relationship between authentic leadership styles and OCBE.

***H3:** Authentic leadership has a significant positive relationship with OCBE*.

#### Supportive Leadership and OCBE

The concept of supportive leadership was introduced and broadened by House, in 1981, with three key components: emotional, informational, and instrumental support. Supportive leadership was narrowed down and defined as “supportive leadership occur when leader expresses concern for, take care of followers' desires and inclinations during making decisions” (Rafferty and Griffin, 2006). Supportive leaders produce an environment of trust, respect, cooperation, and emotional support. Supportive leaders help their followers to resolve the difficult situations they encounter by being open, honest, and fair in interactions (Elsaied, 2019). Emotional, informational, and instrumental support are three components of supportive leadership. Emotional support is when leaders listen to followers' personal and family concerns (McGurk et al., 2014), show sympathy (Rafferty and Griffin, 2006), and try to understand their followers' concerns. Examples of informational support might be demonstrated when army officers share the purpose of upcoming operations with soldiers (McGurk et al., 2014), involving employees in the decision-making process, and sharing the organization's policies. Instrumental support may include, but is not limited to, when a leader allows time and resources to help subordinates complete the tasks that help them achieve a promotion (McGurk et al., 2014).

Schema theory covers the ways people think about the situations they are facing and take steps to mitigate the problems. Their actions are backed by their cognitive-perceptive schemas that represent and exemplify the diversity of awareness and knowledge structures. Furthermore, they organize frameworks for understanding situations or social events and give form and meaning to a domain (Cooper and Shallice, 2006). According to the schema, all the knowledge and experiences are organized into units of information. Schema refers to accepting, understanding, and returning to the behavior of management. With the intellectual maps from these interactive schemas, workforces develop their association with managers (Langenberg and Wesseling, 2016). Previous researchers recognized the status and value of schema theory as a persistent and valuable context for elucidating and organizing knowledge, explaining leadership, and framing analysis of cognitive and intellectual problems in organizations. Employees use these knowledge schemas for scrambling and interpreting information about the supportive behavior of the supervisor. Supportive leadership leads employees to a commitment to organizations and their green policies. Supportive leaders help employees with their environmentally related voluntary behaviors emotionally, knowledge-wise, and instrumentally. The psychological empowerment and emotional support result in the employee's strong commitment to the organization and its green behaviors (Naqvi et al., 2011). Based on the schema theory and academic support, supportive leadership style directly and significantly affects the OCBE. Thus, the following hypothesis can be presumed:

***H4:** Supportive leadership has a significant relationship with OCBE*.

### Mediating Effect of Self-Efficacy

Bandura (1995) defined self-efficacy as “beliefs in one's capabilities to organize and execute courses of action required in managing prospective situations. Efficacy beliefs influence how people think, feel, motivate themselves, and act.” Efficacy belief is the foundation of peoples' motivation; it is a belief in their capabilities as to how they can influence events that will ultimately affect their lives. Saks and Ashforth (2000) argued that general self-efficacy is more likely to impact the workers' behavior in different circumstances by altering personal expectations. Cognitive, motivational, emotional, and selection are the ways through which self-efficacy regulates human behavior (Bandura, 2010). Self-efficacy as a behavior driver has gained the central position in behavioral research (Strzelecka et al., 2018). A leader always guides his followers to act and engage in particular behaviors (Walumbwa et al., 2008; Iqbal et al., 2018; Elsaied, 2019); they can even lead their followers beyond expectations (López-Domínguez et al., 2013). The self-efficacy of followers is connected to their leaders' behavior (Ren and Chadee, 2017). A leader always motivates followers to engage in positive behavior, increases their creativity and productivity, and increases his or her trust in themselves. In this way, leaders enhance followers' beliefs and, ultimately, their self-efficacy (Pillai and Williams, 2004).

Value-belief-norm theory debates that norms of helping pro-social behavior originate from personal values, yet believes that these values are in danger. One can reduce these dangers through action, thus restoring values (Strzelecka et al., 2018). Self-efficacy proponents believe that they are based on situational factors that depend on the surrounding environment. Schutte and Bhullar (2017) argue that negative changeability of environment and related behaviors ignite the motivation, self-efficacy, and self-responsibility, encouraging the person to engage in the activities that can undo these negative happenings. This mainly applies in the case of environmental degradation. The findings of the study of Lauren et al. (2016) advocate that past pro-environmental behavior establishes a sense of being extra skilled and capable of understanding the more difficult environmentally related tasks and challenges in the future. It raises the perception of self-efficacy, and the employee utilizes this for pro-environmental behaviors, for example, OCBE (Jugert et al., 2016).

This same passion for leadership means that the leader provides the foundation for followers to go beyond expectations (Vega-Vázquez et al., 2012) motivating and facilitating for extra-role performance, i.e., pro-environmental and OCBE (López-Domínguez et al., 2013). Followers who have a higher level of self-efficacy are most likely to think they can say they are more involved and committed. They can face difficulties more effectively and can accomplish complex behavior, i.e., OCBE. Lord and Brown (2001) noted that employees' behavior could be formed or influenced by their relationship with their leaders as to the ways these leaders choose to base their contextual effect. According to Podsakoff et al. (1996), self-efficacy is connected to the workers' competencies and motivation. It is more likely to impact the relationships that improve capability and encouragement. Eventually, it plays a moderating role among leadership styles and workers' involvement related to environmental behaviors. Therefore, we hypothesize that:

***H5:** Self-efficacy mediates the relationship between responsible leadership and OCBE*.

***H6:** Self-efficacy mediates the relationship between inclusive leadership and OCBE*.

***H7:** Self-efficacy mediates the relationship between authentic leadership and OCBE*.

### Psychological Ownership

Psychological ownership is a state of mind in which a person develops feelings of ownership for their target. According to Pierce et al. (2001), the central idea of psychological ownership is mentally tied to an object. Psychological ownership is a part of psychological behavior that falls within the domain of positive organizational behavior. Psychological ownership enhances the positivity and endeavor of achievement and success (Avey et al., 2009). It works as a motivator and encourages employees to perform at higher levels (Pierce et al., 2001). Previous studies infer that psychological ownership and employee behavior are positively associated (Mayhew et al., 2007). They further added that the role of psychological ownership strongly influences the in-role and extra-role behaviors of employees. Previous studies indicated that leadership affects employees' psychological behavior (Avey et al., 2009; Bernhard and O'Driscoll, 2011; Kim and Beehr, 2017). Leaders are agents who try to connect employees and organizations and modify the behavior of employees toward organizational success.

On the other hand, cultural perspective by high powers at a distance, and employees' behavior change constrained by their leaders' behavior, is a strong predictor of employees' behavior and psychological ownership in the organization (Hofstede, 1984). The origin of psychological ownership is defined by identifying, and satisfying, basic needs such as home self-efficacy and self-identification (Avey et al., 2009). Responsible, inclusive, authentic, and supportive leadership styles have a significant advantage in meeting these basic needs; this leadership style enhances employees' trust and security. These leaders are also caring about home life and make efforts to satisfy or fulfill employees' needs. Psychological ownership also encourages employees toward organizational effectiveness and creates a strong sense of responsibility for the organization. Therefore, if employees have a sense of psychological ownership of an organization, they may engage in more proactive and positive behaviors. For instance, this could include OCBE. Thus, in this research study, we developed psychological ownership as a mediator between leadership styles and OCBE.

***H8:** Psychological ownership mediates the relationship between supportive leadership and OCBE*.

***H9:** Psychological ownership mediates the relationship between responsible leadership and OCBE*.

***H10:** Psychological ownership mediates the relationship between inclusive leadership and OCBE*.

***H11:** Psychological ownership mediates the relationship between authentic leadership and OCBE*.

## Methodology

### Sample and Procedure

The current research is designed to explain the relationship between leadership styles and OCBE. To examine the direct and mediation associations between responsible, inclusive, authentic, and supportive leadership styles, researchers developed 11 research hypotheses. The current study used a self-administered questionnaire and was distributed among 370 employees of the banking, insurance, pharmaceutical, and teaching service sectors of China. From the distributed questionnaires, we received 320 responses, 300 of which were useful (response rate of 82%), and 20 incomplete responses of which were excluded from the analysis. Data were collected following the free consent of the participants and a brief of the study's objectives. The questionnaire comprised 26 items measuring the different leadership styles, OCBE, self-efficacy, and psychological ownership. Participants of the study were asked to fill out the questionnaire and respond to questions about leadership styles and OCBE, psychological ownership, and self-efficacy. Furthermore, about 76% of respondents were males, and 23% were females; 11.7% of respondents were between 25 and 30 years of age; 70.7% of respondents were between 25 and 30 years of age; 7% were between 30 and 35 years of age, and 10% were above 35 years of age. Moreover, mean values indicated the average responses against each item (ranging from 2.54 to 2.90, SD ranging from 0.95 to 1.145) (see Table 1).

**Table 1:** Descriptive statistics, means, standard deviations, and variance.

**Age**	**Frequency**	**Percent**	**Valid percent**	**Cumulative percent**
20–25	35	11.7	11.7	12.0
25–30	212	70.7	70.7	82.7
30–35	21	7.0	7.0	89.7
Above 35	32	10.3	10.3	100.0
**Gender**	**Frequency**	**Percent**	**Valid percent**	**Cumulative percent**
Male	231	76.7	76.7	77.0
Female	69	23.0	23.0	100.0
Total	300	100.0	100.0	
**Variable**	***N***	**Mean**	**Std. Deviation**	**Variance**
Gender	300	1.226	0.4272	0.183
Age	300	2.153	0.7692	0.592
Responsible leadership	300	2.900	0.9528	0.908
Inclusive leadership	300	2.656	1.058	1.121
Authentic leadership	300	2.643	1.063	1.132
Supportive leadership	300	2.727	1.081	1.169
Psychological ownership	300	2.662	1.145	1.311
Self-efficacy	300	2.540	1.232	1.519
OCBE	300	2.640	1.084	1.175

The questionnaire items were adopted from the previous studies. Responsible leadership was measured by using the scale developed by Voegtlin (2011). Sample item includes “My supervisor involves the affected stakeholders in the decision-making process.” Inclusive leadership was measured by using the scale of Carmeli et al. (2010), composed of three items. Sample item includes “My supervisor is attentive to new opportunities to improve work processes.” Authentic leadership style was measured by using the scale of Walumbwa et al. (2008), consisting of three items. Sample item includes “My supervisor can list his/her three greatest weaknesses.” Moreover, supportive leadership style was measured by using the scale of Rafferty and Griffin (2004) consisting of three items, Sample item includes “My supervisor considers my personal feelings when implementing actions that will affect me.”

OCBE was measured by using the scale developed by Boiral and Paillé (2012). This scale consists of five items, Sample item includes “I actively participate in environmental events organized by my company.” Psychological ownership was measured by using the scales developed by Pierce et al. (1991). Sample item includes “For me, the organization is home.” Self-efficacy was measured with four items on the scale adopted from Sherer et al. (1982). Sample item includes “When I make plans, I am certain I can make them work.” All the conceptual variables were measured on a 5-point Likert scale (1 = strongly agree, 2 = agree, 3 = neutral, 4 = disagree, 5 = strongly disagree). Appendix 1 shows the complete questionnaire.

### Measurement and Structural Models

The measurement models were developed to test the convergent and discriminant validity of the scales. Convergent validity aims to investigate whether the items measure the same concept or not. It consists of the average variance extracted (AVE) and composite reliability (CR). According to Hair et al. (2017), the AVE > 0.5 and CR > 0.70 are accepted. Simultaneously, discriminant validity was checked by the Fornell–Larcker criterion, i.e., taking the square root of AVE. The structural measurement model was developed to test the hypotheses, *P*-values, confidence intervals, β-values, and *t*-statistics, which were all calculated.

### Data Analysis Tools

Data analysis was carried out through AMOS-SEM, a widely used statistical technique in social and management studies. Descriptive and inferential statistics were used in the preliminary analysis stage. All the ethics of the study were taken into consideration, while data were kept confidential and respondents were given enough time to answer the questionnaire honestly.

## Results

The study hypothesis was tested by the means of a structural equation model with maximum-likelihood estimation, using Amos 25 (Brown and Treviño, 2006). First, we investigated the fitness of the measurement model using confirmatory factor analysis (CFA). Before further investigation, we checked the normality and reliability outliers. Some missing values have been removed. We calculated the CR, AVE, and factor loadings of each factor, summing by the average score to measure the level of authentic, supportive, and inclusive leadership; psychological ownership; responsible leadership; and OCBE. All values were found to be within the acceptable range (threshold 0.70), and CR was also above the acceptable threshold (i.e., >0.70) (see Table 2).

**Table 2:** Composite reliability, Cronbach's alpha, average variance extracted, and discriminant validity among study variables.

	**Cronbach's Alpha**	**rho A**	**CR**	**AVE**	**DV**	**MS V**	**AS V**
Authentic leadership	0.721	0.769	0.845	0.648	0.805	0.56	0.35
Supportive leadership	0.875	0.885	0.923	0.76	0.894	0.69	0.63
Inclusive leadership	0.83	0.801	0.882	0.715	0.845	0.55	0.50
Psychological ownership	0.839	0.873	0.893	0.68	0.825	0.59	0.44
Responsible leadership	0.85	0.884	0.909	0.769	0.877	0.72	0.59
Self-efficacy	0.896	0.911	0.928	0.765	0.875	0.76	0.69
Organizational citizenship behavior for environment	0.887	0.904	0.919	0.696	0.834	0.61	0.41

*CR, composite reliability; AVE, average variance extracted; DV, discriminant validity; MSV, maximum shared squared variance; ASV, average shared squared variance*.

Table 2 also represents the Cronbach's alpha, rho A, CR, AVE, discriminant validity, MSV, and ASV. Cronbach's alpha of all the items and CR values range from 0.74 to 0.91 for each variable. Thus, all the internal consistencies meet the standard 0.70 criterion (Nunnally, 1978; Bagozzi and Yi, 1988). AVE is >0.5, but it is acceptable up to 0.4. Fornell and Larcker believed that if AVE is >0.5, but CR is more significant if >0.6, the convergent validity of the construct is still acceptable (Fornell and Larcker, 1981). The results of item loadings, as shown in Table 3, show that the item loadings of all constructs (i.e., responsible, inclusive, authentic, and supportive leadership, psychological ownership, self-efficacy, and OCBE) are in an acceptable range. As mentioned below in the table of statistics, standard loadings of each construct show an estimate of standardized factor loadings >0.70, CR of authentic leadership loading is 0.90, and the AVE is 0.06. The square root of the average variance is 0.078. Table 3 indicates CFA we conducted to examine the construct validity and model fitness.

**Table 3:** Factor loadings and convergent validity results.

**Variable**	**Indicators**	**S/L**	**SSL**	**SSSL**	**No I**	**AVE**	**AV/D V**	**VIF**
Responsible leadership	RL 1	0.830	0.68	2.3	3	0.76	0.76	2.2
	RL 2	0.876	0.76					2.3
	RL 3	0.922	0.85					2.5
Inclusive leadership	IL 1	0.83	0.69	2.1	3	0.71	0.84	1.6
	IL 2	0.84	0.70					1.6
	IL 3	0.86	0.74					1.9
Authentic leadership	AL 1	0.77	0.59	1.9	3	0.64	0.80	1.7
	AL 2	0.92	0.85					2.2
	AL 3	0.70	0.49					1.4
Supportive leadership	SL 1	0.87	0.76	2.3	3	0.76	0.79	1.7
	SL 2	0.89	0.79					2.2
	SL 3	0.91	0.83					2.4
Self- efficacy	SE 1	0.79	0.63	3.0	4	0.76	0.87	1.8
	SE 2	0.82	0.68					2.1
	SE 3	0.91	0.83					2.3
	SE 4	0.95	0.91					2.5
Psychological ownership	PO 1	0.67	0.54	2.7	4	0.68	0.82	1.4
	PO 2	0.88	0.77					2.8
	PO 3	0.92	0.86					3.5
	PO 4	0.79	0.63					1.7
Organizational citizenship behavior for environment	OCBE 1	0.92	0.86	2.7	5	0.69	0.74	3.4
	OCBE 2	0.91	0.84					3.6
	OCBE 3	0.73	0.53					1.9
	OCBE 4	0.72	0.52					1.8
	OCBE 5	0.84	0.71					2.7

*Indicators, standardized loadings, the sum of square standardized loadings, average variance extracted (AVE), number of indicators, RL, responsible leadership; AL, authentic leadership; IL, inclusive leadership; SL, supportive leadership; SE, self-efficacy; PO, psychological ownership OCBE, organizational citizenship behavior for the environment; S/L, standard loadings; SSL, square standardized loadings; SSSL, sum of square standardized loadings; NOI. number of indicators; AVE, average variance extracted; VIF, variance inflation factor*.

Table 3 indicates the results for validity and reliability estimates based on CFA. To fulfill convergent validity rules, AVE should be >0.5. To fulfill the discriminant validity standards, MSV should be less than AVE, AVE should be greater than ASV, and the square root of AVE should be greater than inter-construct correlations. Hence, the AVE value of discriminant validity has been fulfilled. Table 3 also represents the factor loading of each construct, and its items indicated that the item's total correlation values were found to be >0.5 (Hair et al., 2017). Thus, the reliability of all the items of each scale was established. Multicollinearity was measured by variance inflation factors (VIFs) and tolerance. A VIF value of more than 4.0, or tolerance of <0.2, means there is an issue of multicollinearity (Hair et al., 2010). Table 3 reports that all values of VIF show <4.0, which indicates that there is no multicollinearity.

Moreover, CFA was also performed to check the overall goodness of fit of the model. The goodness of fit indices for all the factors (i.e., responsible, inclusive, authentic, and supportive leadership; self-efficacy; and OCBE) indicated the adequacy of the overall model with all its constructs (*X*^2^ = 1011.729; df, 15, *p* < 0.00, CFI 0.931, NFI, 0.920, TLI, 0.903). The value of RMSEA >0.08 indicates that the model is not fit, but in the instance study, the value of RMSEA is 0.05 in Model 1 and 0.07 in Model 2. The results indicated that the M1 hypothesized measurement model is better than M2 (see Table 4).

**Table 4:** Summary of model fit indices.

**Model**	***X*^**2**^**	**Df**	***P***	**CFI**	**NNFI**	**TLI**	**RMSEA**	**Model comparison**	**Δ*χ*^**2**^**
(M1) Two-factor model	1011.729	151	<0.001	0.931	0.920	0.903	0.05		
(M2) One-factor model	2281.430	230	<0.001	0.879	0.868	0.843	0.07	M2-M1	1269.70

*X^2^, chi-square; Df, degree of freedom; CFI, comparative fit index; NNFI, non-normed fit index; TLI, Tucker–Lewis index; RMSEA, root'/mean square error of approximation*.

*Significant at p < 0.001*.

*Thus, the results of preliminary analysis enable the researchers to continue to further test the research hypotheses*.

### Hypothesis Testing

Results of hypotheses testing are shown in Table 5. At the *p* < 0.001, all the direct hypotheses were supported. Results indicated that there is a significant association between leadership styles and OCBE. All the hypotheses tested the best-fitted direct models. The results presented in the table indicated that all the variables have a significant relationship with OCBE. The results indicated that responsible leadership significantly correlated with OCBE (β = 0.32; *p* < 0.001), providing strong support for H1. Results also showed that inclusive leadership is significantly related to OCBE with (β = 0.18; *p* < 0.001) strongly supporting H. To test the significance of the direct relationship between authentic leadership and OCBE, the direct relationship was examined and found significant (β = 0.49; *p* < 0.001). Furthermore, supportive leadership has a significant relationship with OCBE, which is statistically proven (β = 0.43; *p* < 0.001), supporting H4.

**Table 5:** Hypothesis testing results.

**Direct relationship**	**β-value**	**Std. Dev**	***T*-value**	***P*-value**	**Decision**
Responsible leadership -> OCBE	0.324	0.039	8.217	0.000	H1 accepted
Inclusive leadership -> OCBE	0.185	0.052	3.535	0.000	H2 accepted
Authentic leadership -> OCBE	0.49	0.44	3.109	0.000	H3 accepted
Supportive leadership -> OCBE	0.432	0.054	8.031	0.000	H4 accepted
**Path analysis with self-efficacy**					
Responsible leadership -> self-efficacy	0.293	0.085	3.457	0.001	Path analysis
Inclusive leadership -> self-efficacy	0.202	0.055	3.641	0.000	Path analysis
Authentic leadership -> self-efficacy	0.463	0.080	5.804	0.000	Path analysis
Self-efficacy -> OCBEs	0.144	0.051	2.840	0.005	Path analysis
**Path analysis with psychological ownership**					
Supportive leadership -> psychological ownership	0.616	0.043	14.151	0.000	Path analysis
Responsible leadership -> psychological ownership	0.359	0.042	8.445	0.000	Path analysis
Authentic leadership -> psychological ownership	0.329	0.042	7.837	0.000	Path analysis
Inclusive leadership -> psychological ownership	0.475	0.047	10.029	0.000	Path analysis
Psychological ownership -> OCBE	0.346	0.059	5.829	0.000	Path analysis
Self-efficacy -> psychological ownership	0.141	0.049	2.890	0.004	Path analysis

N = 300; *p < 0.05; **p < 0.01; ***p < 0.001.

Thus, all the results suggested that all independent variables have significant relationships with OCBE. Furthermore, a supplementary analysis was carried out to understand how the mediation chain works in causal inferences (Schwab et al., 2011). Similarly, we also found significant results, which supported our hypothesis.

### Mediation Analysis

Mediation analysis was carried out to test the mediating role of self-efficacy and psychological ownership in the association between leadership styles and OCBE. For this purpose, different structural models were developed and tested through AMOS-SEM. Results indicated in Table 6 show the mediation effects of self-efficacy and psychological ownership. H5 of the study supports the mediating role of self-efficacy between responsible leadership and OCBE. After testing this direct relationship, we establish that the value of responsible leadership and OCBE is still a significant/mediating hypothesis. Furthermore, we linked a full mediation model to a partial mediation model to probe whether there is any substantial variation in model fit, with or without the direct path from responsible leadership to OCBE. The process of mediation has some assumptions suggested by Baron and Kenny (1986). These include (a) the independent variable should be associated with the outcome variable, (b) the independent variable must be related to the mediating variable, (c) the mediator should be related to the outcome variable, and (d) if the predictor–outcome path is non-significant, there is complete mediation. If it is significant, there is partial mediation. Bootstrapping was performed using 2,000 resamples to test the significance of the indirect effect (Hayes, 2009). We tested (β = 0.042; *p* < 0.029). Through the path analysis, the results are still significant that there is a partial mediating role between self-efficacy and responsible leadership and OCBE. H6 indicated the mediating role of self-efficacy between inclusive leadership and OCBE, so, through the above approach, we found significant results during the analysis. We found (β = 0.029; *p* < 0.030). The value is substantial; self-efficacy plays a partial mediation role between inclusive leadership and OCBE. H7 was tested to examine the mediating role of self-efficacy between authentic leadership style and OCBE. We found (β = 0.067; *p* < 0.012) results, which indicate the partial mediation. Based on the direct links among variables, we carried out additional analyses to help in understanding how the mediation chain works in causal inferences (Schwab et al., 2011). When the direct path is not significant, the significant indirect path is categorized as indirect-only mediation or complete mediation (Schwab et al., 2011).

**Table 6:** Specific indirect effect.

**Path**	**β-value**	**Std. Dev**	***T*-value**	***P*-value**	**Decision**
Responsible leadership -> self-efficacy -> OCBE	0.042	0.019	2.183	0.029	H5 accepted
Inclusive leadership -> self-efficacy -> OCBE	0.029	0.013	2.178	0.030	H6 accepted
Authentic leadership -> self-efficacy -> OCBE	0.067	0.027	2.503	0.012	H7 accepted
Supportive leadership -> psychological ownership-> OCBE	0.213	0.041	5.173	0.000	H8 accepted
Responsible leadership -> psychological ownership-> OCBE	0.124	0.022	5.713	0.000	H9 accepted
Inclusive leadership -> psychological ownership -> OCBE	0.164	0.030	5.493	0.000	H10 accepted
Authentic leadership-> psychological ownership -> OCBE	0.114	0.025	4.492	0.000	H11 accepted

N = 300; *p < 0.05; **p < 0.01; ***p < 0.001.

In this study, we developed H8 and tested a model that sought to better understand the effect of the well-indicated psychological behavior of employees, which mediates between supportive leadership and OCBE. We found (β = 0.021; *p* < 0.000). Thus, this research hypothesis supported a partial mediation because the direct effect was mentioned in the above table; there the relationship was significant with a *p*-value of <0.001. Furthermore, after mediator psychological ownership, there is no high effect of the path so psychological ownership mediates partially. Similarly, H9 predicted the mediating role of psychological ownership between responsible leadership style and OCBE. We found significant results (β = 0.12; *p* < 0.000), which shows the partial mediation; thus, H9 is accepted. H10 was tested to examine the mediating role of psychological ownership between inclusive leadership style and OCBE. We found (β = 0.16; *p* < 0.000) as we tested the already-established direct effect in Table 5 and we saw a significant relationship between inclusive leadership and OCBE. After the mediator, the results are still significant so our H10 is accepted; there is a partial mediation. H11 predicted the mediating role of psychological ownership between authentic leadership style and OCBE. We found (β = 0.11; *p* < 0.000). All values supported our research hypothesis regarding psychological ownership, which partially mediates the relationship between leadership styles and OCBE.

Therefore, these findings or statistics support how leadership style affects OCBE and how self-efficacy, or employees and psychological ownership, mediates the relationship between leadership styles and OCBE. Table 6 indicates the results are still significant when we enter a mediator in the analysis, so the results are still significant; thus, there is a partial mediation.

## Discussion

This research study investigated the mechanism that illuminates how different leadership styles (i.e., responsible, inclusive, authentic, and supportive) enhance the OCB within Chinese organizations' environments. We tested a series of hypotheses. Using AMOS-SEM, we examined the mediating role of self-efficacy and psychological ownership. The statistical results confirm that self-efficacy and psychological ownership positively mediates the relationship between leadership styles and OCBE. By answering the calls of previous studies (Zhang et al., 2016; Robertson and Barling, 2017; Priyankara et al., 2018; Han et al., 2019b), the findings of this study indicate that leaders' environmental support has a positive impact on employees' OCBE. The positive effect of a leader's support for the environment through OCBE is consistent with the findings of Raineri and Paillé (2016), who studied French business schools and found that leadership is positively associated with employees' OCBE. Building upon the characteristics of responsible leadership, this study established that responsible leadership behavior is closely associated with OCBE, and the findings of this study are aligned and accede that responsible leadership behavior is significantly and positively associated with OCBE. Responsible leaders safeguard stakeholders' interests, both organizational and communal, including environmental concerns, become socially responsible, and protect the ecological interests of employees and society (Han et al., 2019b; Zhao and Zhou, 2019). These findings are also aligned with the study of Afsar et al. (2020), who found that responsible leaders support the environmentally friendly behavior of employees and encourage them to reduce energy consumption, save paper, and assist colleagues in green practices. Furthermore, inclusive leaders encourage and include employees and followers in the organization's affairs by enhancing their sense of belonging and identification with the organization. Inclusive leadership is distinctive from other leadership styles as it allows employees to participate in organizational affairs (Sugiyama et al., 2016). This unique characteristic encourages employees to express behavior that is beneficial for the organization as well as society. The optimal distinctiveness theory also advocates distinctiveness, which encourages employees to engage in distinctive behaviors. The findings of the current study also support that decision-making encourages employees to engage in friendly behaviors, i.e., OCBE. These findings align with the previous research of Javed et al. (2017).

Likewise, authentic leadership encourages followers to act according to their values, feelings, beliefs, strengths, and weaknesses (Walumbwa et al., 2008; Iqbal et al., 2018). The constructive moral perspective of authentic leadership develops employees' vital attitude at the workplace, which inspires employees to engage in voluntary constructive behavior that is beneficial for the workplace and society, i.e., OCBE (Iqbal et al., 2018). This study also proved empirically, and acceded to, previous studies' similar findings (Iqbal et al., 2018; Liu et al., 2018) that authentic leadership is positively associated with OCBE. The supportive leadership style considers followers' desires and inclinations while making decisions, and is also regarded as responsible for employees' proactive, positive behaviors (Rafferty and Griffin, 2006). The environment of trust, respect, emotional support, and cooperation is produced in the shadow of supportive leadership behavior. This cooperation and emotional support help employees resolve complex problems, be open and honest, and interact freely with organizational and society-related issues, i.e., environmental issues. The current study concludes that emotional, informational, and instrumental support helps employees engage with positive behaviors, i.e., OCBE, aligning with the findings of previous studies (Rafferty and Griffin, 2006; Elsaied, 2019).

Furthermore, the study entails a mediation mechanism of self-efficacy and psychological ownership of employees, in connection to leadership styles and OCBE, which enhances employees' environmental performance and is also considered vital for environmental concerns and sustainability. The leader guides his followers to engage in particular positive behaviors. The self-efficacy of followers, connected with the leader's behaviors, magnifies these behaviors (Ren and Chadee, 2017), increasing his creativity and trust in himself. Employees experiencing a higher level of self-efficacy are most likely to consider themselves more important, involved, and committed. They can face difficulties more effectively and can accomplish challenging voluntary behavior.

Similarly, the current study's findings verified empirically that the mediating role of self-efficacy is positive with responsible, inclusive, authentic, and supportive leadership styles for OCBE. Being a state of mind, psychological ownership develops feelings of belonging toward the target object, sharing positivity, and endeavoring for achievement and success (Avey et al., 2009). As a motivator, it encourages employees to perform at higher levels. Leaders as an agent connect employees and organizations by motivating employees' psychological ownership, thus modifying the behavior of employees toward organizational success. Psychological ownership also encourages employees toward organizational effectiveness and creates a strong sense of responsibility for the organization. The current study used psychological ownership as a mediator for leadership styles and OCBE. If employees have a sense of psychological ownership of the organization, they may engage in more proactive and positive behaviors, for example, OCBE. The valuable findings of the study practically proved that psychological ownership mediates the relationship between responsible, inclusive, authentic, and supportive leadership behaviors and OCBE. This is also aligned with the findings of previous studies (Bernhard and O'Driscoll, 2011; Kim and Beehr, 2017). The above discussion concludes that OCBE is significantly associated with different leadership styles (responsible, inclusive, authentic, supportive) of Chinese banking, insurance, pharmaceutical, and teaching service employees. Moreover, self-efficacy and psychological ownership also mediate this relationship.

## Conclusion

The current study aimed to analyze the effects of multiple leadership styles, i.e., responsible, inclusive, authentic, and supportive, connected with OCBE. Self-efficacy and psychological ownership were introduced as a mediator in the above relationship. AMOS-SEM was used to analyze the data collected from the employees in China's service and manufacturing sectors. The empirical results clarify that responsible, inclusive, authentic, and supportive leadership styles are positively and significantly associated with employee's OCBE. Positive organizational behavior advocates employees' proactive behavior targeted toward the organization, as well as toward society. The study identified the positive role of leaders to facilitate employees' OCBEs from a multiple leadership styles approach. Furthermore, the introduction of self-efficacy and psychological ownership as a mediator was also investigated with OCBE. The empirical results of this study explained that as the level of self-efficacy and feelings of psychological ownership is amplified among the employees, their level of OCBE also increased.

### Managerial Implications

The current research study has considerable implications for managers and policymakers, and, in different ways, for human resource development practitioners. First, this research study indicated statistical findings that leadership styles tend to bring out OCBE. Leadership style helps employees change their perception of the environment and be more responsible and proactive for environmental-saving behaviors. Furthermore, OCBE is an essential consequence of an employee's performance and well-being. Therefore, by hiring responsible, inclusive, authentic, and supportive leaders, and providing adequate training to existing leaders, the trait of these leaders can be utilized to make employees more capable of exercising OCBE and change the perception of employees' values. Second, employees' self-efficacy, feelings, and trust regarding environmental issues play a vital role in developing environmentally protective behaviors. The management and policymakers should formulate such policies, and indulge employees in such training, that enhance employees' self-efficacy levels toward the environment. Third, the current study draws attention to psychological ownership. It also clarifies how leadership styles enhance employee OCBEs through psychological ownership. Furthermore, it offers a unique, theoretical lens to elaborate employee OCBEs by accompanying existing approaches from the social exchange, self-determination, and theory of planned behavior.

### Theoretical Contributions

The findings of this study make important contributions to the literature. First, this research study indicates the relationship of leadership styles such as responsible leadership, inclusive leadership, authentic leadership, and supportive leadership with OCBE. Social learning theory suggests that human behavior is primarily learned through observation; thus, in this way, both direct and indirect learning occurs. Social learning theory argued that individuals could learn through their behavior by observing others. Social learning theory also emphasizes that employees learn through focusing on a role model and adopting behavior by discovering what action is rewarded (Bandura, 1986). This research study contributes to how leaders' support leads to OCBE. Theoretically speaking, the social learning theory supports an explanation of the complex mechanism of leadership, particularly responsible leadership with OCBE.

The responsible leader takes additional care of the interest of the stakeholders, exercises his effect on them, and encourages employees, thus influencing employees' values and self-assessment toward leaders and identification with the organization. When employees identify themselves with leaders and organizations, observing leaders and organizational environmental concerns, they are influenced and agree with the norms and values of leaders. They also act on behalf of leaders for sustainable development of the organization and environmental protection (Paillé et al., 2014). Responsible leaders strive to integrate and protect all the stakeholders' interests and benefits, particularly their ecological interests. According to the scope of social identity theory, this helps employees to engage in the OCBE. In such cases, responsible leadership and leader identification can be good explanatory mechanisms. Furthermore, in theoretical contribution, optimal distinctiveness theory, an extension of social identity theory, discusses that individuals always need to be both different from, and similar to, others simultaneously. This study also contributed to how inclusive leadership influences OCBE. The conceptualization of inclusion is viewed as the individual's need for belonging and uniqueness (Randel et al., 2018). It facilitates a sense of belonging within the organization and its policies. The current study investigates the unique framework and its interpretation to elaborate on the relationship between inclusive leadership and OCBE. Inclusive leaders include employees in the discussion and decision-making process and value their voice. The decision-making process, and inclusion in the organizational discussion, encourages environmentally oriented employees to promote and implement new ideas and establish new practices to save the environment. From the perspective of innovation and environmentally friendly behavior, employees need the support of organizations that they find in the shape of inclusive leadership (Javed et al., 2017). Specifically, through the theorizing and demonstration of constructive effects of inclusive leadership on OCBE, this study supports interactions between inclusive leadership and its impact on employees' citizenship behavior for the environment.

This study further contributed to four components of authentic leadership and OCBE. The leaders' self-awareness of identity, values, emotions, and goals (i.e., environmental goals and values) influences their traits which, subsequently, influence the employees. The findings of this study maintain the idea that the impact of authentic leadership style on OCBE of employees is stronger when these behaviors are objective and directed toward the organization or society, i.e., environment, compared to when they are directed toward individuals. Additionally, schema theory assertions are the ways of considering employees' situations and taking steps to mitigate their problems. Employees' actions are backed by their cognitive perceptive schemas that represent and exemplify the diversity of awareness and knowledge structures, organize frameworks for understanding situations or social events, and give form and meaning to a domain (Cooper and Shallice, 2006). Previous researchers recognized the schema theory as a valuable context for organizing knowledge, explaining leadership, and enclosing analysis of cognitive and intellectual problems in organizations. Employees use this knowledge schema for analyzing and interpreting information about the supportive behavior of leaders. Supportive leadership leads employees to a commitment to organizations and their green policies. Supportive leaders help employees with their environmentally related voluntary behaviors emotionally, knowledge-wise, and instrumentally. Supportive leaders produce an environment of trust, respect, psychological empowerment, and emotional support (Naqvi et al., 2011). The psychological empowerment and emotional support result in an employee's strong commitment to the organization and its green behaviors, and employees exert OCBE.

Finally, this study contributed to the value-belief-norm theory as a norm of helping pro-social behavior originate from personal values, and believes that these values are in danger. One should reduce these dangers through proactive action and restore values (Strzelecka et al., 2018). A leader's motivation for their followers' positive behavior increases his or her creativity and productivity and increases his or her trust in themselves, or, in other words, increases his self-efficacy. Self-efficacy beliefs are based on situational factors that depend on the surrounding environment. Negative environmental changes ignite employees' motivation, self-efficacy, and self-responsibility and encourage them to engage in the activities that can undo these negative happenings. This particularly applies to environmental degradation. Pro-environmental behavior establishes the sense of being extra-skilled and capable of facing the more difficult environmentally related tasks and challenges in the future. Thus, we contributed that this will raise the perception of self-efficacy, and employees will utilize this for pro-environmental behaviors, for example, OCBE.

### Limitations and Future Directions

There are some limitations in the present study, but the limitations can serve as avenues for future research. First, this study is cross-sectional and obtained data at one point in time. Researchers are encouraged to pursue a longitudinal examination, multi-sourcing data to explore the causality of the relationships between multiple leadership styles and OCBE. Furthermore, causality may be established by following a quasi-experimental approach that could improve the findings. Third, other negative leadership styles, i.e., abusive leadership, should be tested with OCBE. Finally, the impact of other green human resource management practices, i.e., green hiring and green performance, should be seen in the magnitude of the relationship between leadership and OCBE.

## Data Availability Statement

The datasets generated for this study are available on request to the corresponding author.

## Ethics Statement

Ethical review and approval was not required for the study on human participants in accordance with the local legislation and institutional requirements. Written informed consent for participation was not required for this study in accordance with the national legislation and the institutional requirements.

## Author Contributions

IU contributed to the data curation, formal analysis, and original draft and revision of the manuscript. WW contributed to the revision of the manuscript. HW contributed to the supervision and guidelines. SMAS contributed to the review and formatting of the manuscript. AA contributed to the, conceptualization, formal analysis, and original draft and revision of the manuscript. SM contributed to the writing, review and editing of the manuscript.

## Conflict of Interest

The authors declare that the research was conducted in the absence of any commercial or financial relationships that could be construed as a potential conflict of interest.

## Appendix

**Table A1:** Formal questionnaire.

**Responsible leadership**
My supervisor involves the affected stakeholders in the decision-making process.
My supervisor tries to achieve a consensus among the affected stakeholders.
My supervisor weighs different stakeholder claims before making a decision.
**Inclusive leadership**
My supervisor is attentive to new opportunities to improve work processes.
My supervisor is available for consultation on problems.
My supervisor is accessible for discussing emerging problems.
**Authentic leadership**
My supervisor can list his/her three greatest weaknesses.
My supervisor lets others know who he/she truly is as a person.
My supervisor admits my mistakes to others.
**Supportive leadership**
My supervisor considers my personal feelings when implementing actions that will affect me.
My supervisor takes into account my personal needs
My supervisor ensures the interests of employees are considered when making decisions
**Psychological ownership**
“For me,” the organization is home
I am totally comfortable being in this organization
I feel that I belong in this organization
I feel that this organization's success is my success.
**Self-efficacy**
When I make plans, I am certain I can make them work
If something looks too complicated, I will not even bother to try it
If I can't do a job the first time, I keep trying until I can
I avoid facing difficulties
**Organizational citizenship behavior for the environment**
I actively participate in environmental events organized by my company
I voluntarily participate in environmental events outside the organization to contribute to the image of the organization
I spontaneously give my time to remind colleagues to pay attention to environmental protection at work
I make suggestions to my colleagues about ways to protect the environment more effectively
I shall persuade the company or colleagues to buy environmental products

## References

[B1] Afsar B. Maqsoom A. Shahjehan A. Afridi S. A. Nawaz A. Fazliani H. (2020). Responsible leadership and employee's proenvironmental behavior: the role of organizational commitment, green shared vision, and internal environmental locus of control. Corporate Soc. Responsibil. Environ. Manage. 27, 297–312. 10.1002/csr.1806

[B2] Alt E. Spitzeck H. (2016). Improving environmental performance through unit-level organizational citizenship behaviors for the environment: a capability perspective. J. Environ. Manage. 182, 48–58. 10.1016/j.jenvman.2016.07.034 27454096

[B3] Avey J. B. Avolio B. J. Crossley C. D. Luthans F. (2009). Psychological ownership: theoretical extensions, measurement and relation to work outcomes. J. Org. Behav. Int. J. Indus. Occup. Org. Psychol. Behav. 30, 173–191. 10.1002/job.583

[B4] Avolio B. J. Gardner W. L. (2005). Authentic leadership development: getting to the root of positive forms of leadership. Leadersh. Q. 16, 315–338. 10.1016/j.leaqua.2005.03.001

[B5] Bagozzi R. P. Yi Y. (1988). On the evaluation of structural equation models. J. Acad. Marketing Sci. 16, 74–94. 10.1007/BF02723327

[B6] Bandura A. (1986). Social Foundations of Thought and Action. Englewood Cliffs, NJ.

[B7] Bandura A. (1995). Exercise of personal and collective efficacy in changing societies. Self Efficacy Changing Soc. 15:334. 10.1017/CBO9780511527692.003

[B8] Bandura A. (2010). Self-efficacy-Bandura,” in The Corsini Encyclopedia of Psychology, 13. 10.1002/9780470479216.corpsy0836

[B9] Bansal P. Song H.-C. (2017). Similar but not the same: differentiating corporate sustainability from corporate responsibility. Acad. Manage. Ann. 11, 105–149. 10.5465/annals.2015.0095

[B10] Baron R. M. Kenny D. A. (1986). The moderator–mediator variable distinction in social psychological research: conceptual, strategic, and statistical considerations. J. Pers. Soc. Psychol. 51:1173. 10.1037/0022-3514.51.6.1173 3806354

[B11] Bernhard F. O'Driscoll M. P. (2011). Psychological ownership in small family-owned businesses: leadership style and nonfamily-employees' work attitudes and behaviors. Group Org. Manage. 36, 345–384. 10.1177/1059601111402684

[B12] Boiral O. Paillé P. (2012). Organizational citizenship behaviour for the environment: measurement and validation. J. Business Ethics 109, 431–445. 10.1007/s10551-011-1138-9

[B13] Brown M. E. Treviño L. K. (2006). Ethical leadership: a review and future directions. Leadersh. Q. 17, 595–616. 10.1016/j.leaqua.2006.10.004

[B14] Carmeli A. Reiter-Palmon R. Ziv E. (2010). Inclusive leadership and employee involvement in creative tasks in the workplace: the mediating role of psychological safety. Creat. Res. J. 22, 250–260. 10.1080/10400419.2010.504654

[B15] Chan E. S. Hsu C. H. (2016). Environmental management research in hospitality. Int. J. Contemporary Hospital. Manage. 28, 886–923. 10.1108/IJCHM-02-2015-0076

[B16] Conchie S. M. Taylor P. J. Donald I. J. (2012). Promoting safety voice with safety-specific transformational leadership: the mediating role of two dimensions of trust. J. Occup. Health Psychol. 17:105. 10.1037/a0025101 21875211

[B17] Cooper R. P. Shallice T. (2006). Hierarchical schemas and goals in the control of sequential behavior. Psychol. Rev. 113, 887–916. 10.1037/0033-295X.113.4.887 17014307

[B18] Daily B. F. Bishop J. W. Govindarajulu N. (2009). A conceptual model for organizational citizenship behavior directed toward the environment. Business Soc. 48, 243–256. 10.1177/0007650308315439

[B19] Elsaied M. (2019). Supportive leadership and EVB. J. Manage. Dev. 38, 225–237. 10.1108/JMD-04-2018-0119

[B20] Felin T. Foss N. J. Ployhart R. E. (2015). The microfoundations movement in strategy and organization theory. Acad. Manag. Ann. 9, 575–632. 10.1080/19416520.2015.1007651

[B21] Fornell C. Larcker D. F. (1981). Structural Equation Models With Unobservable Variables and Measurement Error: Algebra and Statistics. Los Angeles, CA: Sage Publications.

[B22] Galpin T. Whittington J. L. (2012). Sustainability leadership: from strategy to results. J. Business Strategy. 33, 40–48. 10.1108/02756661211242690

[B23] Goleman D. (2004). What makes a leader? Harvard Bus. Rev. 82, 82–91. 10187249

[B24] Hair J. F. Black W. C. Babin B. J. Anderson R. E. (2010). Multivariate Data Analysis: A Global Perspective, 7th Edn. Uppersaddle River, NJ: Pearson Prentice Hall.

[B25] Hair J. F. Hult G. T. M. Ringle C. M. Sarstedt M. Thiele K. O. (2017). Mirror, mirror on the wall: a comparative evaluation of composite-based structural equation modeling methods. J. Acad. Market. Sci. 45, 616–632. 10.1007/s11747-017-0517-x

[B26] Han Z. Wang Q. Yan X. (2019a). How responsible leadership motivates employees to engage in organizational citizenship behavior for the environment: a double-mediation model. Sustainability 11:605. 10.3390/su11030605

[B27] Han Z. Wang Q. Yan X. (2019b). How responsible leadership predicts organizational citizenship behavior for the environment in China. Leadership Org. Dev. J. 40, 305–318. 10.1108/LODJ-07-2018-0256

[B28] Hayes A. F. (2009). Beyond Baron and Kenny: statistical mediation analysis in the new millennium. Commun. Monogr. 76, 408–420. 10.1080/03637750903310360

[B29] Hofstede G. (1984). Culture's Consequences: International Differences in Work-Related Values, Vol. 5. Sage.

[B30] Iqbal S. Farid T. Ma J. Khattak A. Nurunnabi M. (2018). The impact of authentic leadership on organizational citizenship behaviours and the mediating role of corporate social responsibility in the banking sector of Pakistan. Sustainability 10:2170. 10.3390/su10072170

[B31] Javed B. Naqvi S. M. M. R. Khan A. K. Arjoon S. Tayyeb H. H. (2017). Impact of inclusive leadership on innovative work behavior: the role of psychological safety–CORRIGENDUM. J. Manage. Org. 23, 472–472. 10.1017/jmo.2017.17

[B32] Jugert P. Greenaway K. H. Barth M. Büchner R. Eisentraut S. Fritsche I. (2016). Collective efficacy increases pro-environmental intentions through increasing self-efficacy. J. Environ. Psychol. 48, 12–23. 10.1016/j.jenvp.2016.08.003

[B33] Kim M. Beehr T. A. (2017). Self-efficacy and psychological ownership mediate the effects of empowering leadership on both good and bad employee behaviors. J. Leadership Org. Stud. 24, 466–478. 10.1177/1548051817702078

[B34] Kura K. M. (2016). Linking environmentally specific transformational leadership and environmental concern to green behaviour at work. Global Business Rev. 17, 1S−14S. 10.1177/0972150916631069

[B35] Lamm E. Tosti-Kharas J. King C. E. (2015). Empowering employee sustainability: perceived organizational support toward the environment. J. Business Ethics 128, 207–220. 10.1007/s10551-014-2093-z

[B36] Lamm E. Tosti-Kharas J. Williams E. G. (2013). Read this article, but don't print it: organizational citizenship behavior toward the environment. Group Org. Manage. 38, 163–197. 10.1177/1059601112475210

[B37] Langenberg S. Wesseling H. (2016). Making sense of Weick's organising. A philosophical exploration. Philos. Manage. 15, 221–240. 10.1007/s40926-016-0040-z

[B38] Lauren N. Fielding K. S. Smith L. Louis W. R. (2016). You did, so you can and you will: self-efficacy as a mediator of spillover from easy to more difficult pro-environmental behaviour. J. Environ. Psychol. 48, 191–199. 10.1016/j.jenvp.2016.10.004

[B39] Liu Y. Fuller B. Hester K. Bennett R. J. Dickerson M. S. (2018). Linking authentic leadership to subordinate behaviors. Leadership Org. Dev. J. 39, 218–233. 10.1108/LODJ-12-2016-0327

[B40] López-Domínguez M. Enache M. Sallan J. M. Simo P. (2013). Transformational leadership as an antecedent of change-oriented organizational citizenship behavior. J. Bus. Res. 66, 2147–2152. 10.1016/j.jbusres.2013.02.041

[B41] Lord R. G. Brown D. J. (2001). Leadership, values, and subordinate self-concepts. Leadersh. Q. 12, 133–152. 10.1016/S1048-9843(01)00072-8

[B42] Mayhew M. G. Ashkanasy N. M. Bramble T. Gardner J. (2007). A study of the antecedents and consequences of psychological ownership in organizational settings. J. Soc. Psychol. 147, 477–500. 10.3200/SOCP.147.5.477-500 18225830

[B43] McGurk D. Sinclair R. R. Thomas J. L. Merrill J. C. Bliese P. D. Castro C. A. (2014). Destructive and supportive leadership in extremis: relationships with post-traumatic stress during combat deployments. Military Behav. Health 2, 240–256. 10.1080/21635781.2014.963765

[B44] Naqvi I. H. Bokhari S. H. A. Aziz S. (2011). The impact of human resource (HR) performance management on project outcome. Afr. J. Bus. Manag. 5, 8491–8499. 10.5897/AJBM11.665

[B45] Nembhard I. M. Edmondson A. C. (2006). Making it safe: the effects of leader inclusiveness and professional status on psychological safety and improvement efforts in health care teams. J. Org. Behav. 27, 941–966. 10.1002/job.413

[B46] Nunnally J. C. (1978). Psychometric Theory, 2nd Edn. Hillsdale, NJ: McGraw-Hill.

[B47] Paillé P. Boiral O. Chen Y. (2013). Linking environmental management practices and organizational citizenship behaviour for the environment: a social exchange perspective. Int. J. Human Resource Manage. 24, 3552–3575. 10.1080/09585192.2013.777934

[B48] Paillé P. Chen Y. Boiral O. Jin J. (2014). The impact of human resource management on environmental performance: an employee-level study. J. Business Ethics 121, 451–466. 10.1007/s10551-013-1732-0

[B49] Paillé P. Mejía-Morelos J. H. (2014). Antecedents of pro-environmental behaviours at work: the moderating influence of psychological contract breach. J. Environ. Psychol. 38, 124–131. 10.1016/j.jenvp.2014.01.004

[B50] Pierce J. L. Kostova T. Dirks K. T. (2001). Toward a theory of psychological ownership in organizations. Acad. Manage. Rev. 26, 298–310. 10.5465/amr.2001.4378028

[B51] Pierce J. L. Rubenfeld S. A. Morgan S. (1991). Employee ownership: a conceptual model of process and effects. Acad. Manage. Rev. 16, 121–144. 10.5465/amr.1991.4279000

[B52] Pillai R. Williams E. A. (2004). Transformational leadership, self-efficacy, group cohesiveness, commitment, and performance. J. Org. Change Manage. 10.1108/09534810410530584

[B53] Pless N. M. Maak T. Stahl G. K. (2011). Developing responsible global leaders through international service-learning programs: the Ulysses experience. Acad. Managem. Learning Educ. 10, 237–260. 10.5465/amle.10.2.zqr237

[B54] Podsakoff P. M. MacKenzie S. B. Bommer W. H. (1996). Transformational leader behaviors and substitutes for leadership as determinants of employee satisfaction, commitment, trust, and organizational citizenship behaviors. J. Manage. 22, 259–298. 10.1177/014920639602200204

[B55] Priyankara H. P. R. Luo F. Saeed A. Nubuor S. A. Jayasuriya M. P. F. (2018). How does leader's support for environment promote organizational citizenship behaviour for environment? A multi-theory perspective. Sustainability 10:271. 10.3390/su10010271

[B56] Rafferty A. E. Griffin M. A. (2004). Dimensions of transformational leadership: conceptual and empirical extensions. Leadersh. Q. 15, 329–354. 10.1016/j.leaqua.2004.02.009

[B57] Rafferty A. E. Griffin M. A. (2006). Refining individualized consideration: distinguishing developmental leadership and supportive leadership. J. Occup. Organ. Psychol. 79, 37–61. 10.1348/096317905X36731

[B58] Raineri N. Paillé P. (2016). Linking corporate policy and supervisory support with environmental citizenship behaviors: the role of employee environmental beliefs and commitment. J. Business Ethics 137, 129–148. 10.1007/s10551-015-2548-x

[B59] Ramus C. A. Steger U. (2000). The roles of supervisory support behaviors and environmental policy in employee “ecoinitiatives” at leading-edge European companies. Acad. Manage. J. 43, 605–626. 10.2307/1556357

[B60] Randel A. E. Galvin B. M. Shore L. M. Ehrhart K. H. Chung B. G. Dean M. A.. (2018). Inclusive leadership: realizing positive outcomes through belongingness and being valued for uniqueness. Human Resource Manage. Rev. 28, 190–203. 10.1016/j.hrmr.2017.07.002

[B61] Ren S. Chadee D. (2017). Ethical leadership, self-efficacy and job satisfaction in China: the moderating role of guanxi. Personnel Rev. 46, 371–388. 10.1108/PR-08-2015-0226

[B62] Robertson J. L. Barling J. (2013). Greening organizations through leaders' influence on employees' pro-environmental behaviors. J. Organ. Behav. 34, 176–194. 10.1002/job.1820

[B63] Robertson J. L. Barling J. (2017). Toward a new measure of organizational environmental citizenship behavior. J. Bus. Res. 75, 57–66. 10.1016/j.jbusres.2017.02.007

[B64] Rooney J. A. Gottlieb B. H. (2007). Development and initial validation of a measure of supportive and unsupportive managerial behaviors. J. Vocat. Behav. 71, 186–203. 10.1016/j.jvb.2007.03.006

[B65] Saks A. M. Ashforth B. E. (2000). Change in job search behaviors and employment outcomes. J. Vocat. Behav. 56, 277–287. 10.1006/jvbe.1999.1714

[B66] Schutte N. S. Bhullar N. (2017). Approaching environmental sustainability: perceptions of self-efficacy and changeability. J. Psychol. 151, 321–333. 10.1080/00223980.2017.1289144 28339352

[B67] Schwab A. Abrahamson E. Starbuck W. H. Fidler F. (2011). Perspective—researchers should make thoughtful assessments instead of null-hypothesis significance tests. Org. Sci. 22, 1105–1120. 10.1287/orsc.1100.0557

[B68] Sherer M. Maddux J. E. Mercandante B. Prentice-Dunn S. Jacobs B. Rogers R. W. (1982). The self-efficacy scale: construction and validation. Psychol. Rep. 51, 663–671. 10.2466/pr0.1982.51.2.663

[B69] Shore L. M. Randel A. E. Chung B. G. Dean M. A. Holcombe Ehrhart K. Singh G. (2011). Inclusion and diversity in work groups: a review and model for future research. J. Manage. 37, 1262–1289. 10.1177/0149206310385943

[B70] Strzelecka M. Woosnam K. M. Nisbett G. S. (2018). Self-efficacy mechanism at work: the context of environmental volunteer travel. J. Sustainable Tourism 26, 2002–2020. 10.1080/09669582.2018.1526297

[B71] Sugiyama K. Cavanagh K. V. van Esch C. Bilimoria D. Brown C. (2016). Inclusive leadership development: drawing from pedagogies of women's and general leadership development programs. J. Manage. Educ. 40, 253–292. 10.1177/1052562916632553

[B72] Temminck E. Mearns K. Fruhen L. (2015). Motivating employees towards sustainable behaviour. Business Strategy Environ. 24, 402–412. 10.1002/bse.1827

[B73] Vega-Vázquez M. Cossío-Silva F. J. Martín-Ruíz D. (2012). Does the firm's market orientation behaviour influence innovation's success? Managem. Decision. 50, 1445–1464 10.1108/00251741211262024

[B74] Voegtlin C. (2011). Development of a scale measuring discursive responsible leadership. J. Bus. Ethics 98, 57–73. 10.1007/s10551-011-1020-9

[B75] Walumbwa F. O. Avolio B. J. Gardner W. L. Wernsing T. S. Peterson S. J. (2008). Authentic leadership: development and validation of a theory-based measure. J. Manage. 34, 89–126. 10.1177/0149206307308913

[B76] Zhang J. Chen Y. Liu J. (2016). Ethical leadership and OCBE: The influence of prosocial motivation and self accountability. Paper presented at the Academy of Management Proceedings (Briarcliff Manor, NY), 10510. 10.5465/ambpp.2016.15588abstract

[B77] Zhao H. Zhou Q. (2019). Exploring the impact of responsible leadership on organizational citizenship behavior for the environment: a leadership identity perspective. Sustainability 11:944. 10.3390/su11040944

